# School Medical Inspection and Its Influences

**Published:** 1918-11-23

**Authors:** 


### LONDON'S HEALTH.

### II. School Medical Inspection and its Influences.

The second part of Dr. Hamer's report * deals with education and gives a full and detailed account of the work of school medical inspection and of the resulting following up and treatment which were carried out during the year. The report contains much important information, and many questions relating to the influences of the war, and of home and school conditions, on the health and welfare of school children are discussed in a very informing and interesting manner. The mind of the reader is at once focussed on the relationship which exists between environment and the health standard of the school child.

### War Conditions and School Health.

In this connection Dr. Hamer points out that the favourable economic conditions at present existing are indicated by the fact that the number of necessitous children receiving free meals continues to decline. Referring to the question of war-bread, he states that there exists no conclusive evidence that it was responsible for the symptoms of illness mentioned by some observers. A much more serious question to which reference is made is the extent to which milk supplied to necessitous school children was found to contain added water. The results of analyses at certain times of the year gave as high as 50 per cent. of the samples containing added water. But notwithstanding this and the existing food difficulties, the result of the special inquiry carried out on the number of malnourished children found on medical examination showed improvement and not deterioration. That the children of London in the fifth year of the war should show improvement in nutrition instead of increased evidence of malnutrition is an extremely significant and encouraging fact. There were two serious interferences with the health of the school children during the year which directly resulted from war conditions. There was a marked increase in the prevalence of scabies, due, no doubt, to infection from returned soldiers, and the results of the treatment of ailments in school children were less satisfactory, due to diminished facilities at certain hospitals and the increased difficulty experienced in "following up."

### Vermin as an Ætiological Factor in Disease.

With reference to verminous conditions Dr. Hamer says "the great improvement in this respect brought about since 1870 and particularly during the last thirty years is appreciated by everyone who has had experience of London children extending over that period, but the campaign against vermin has, unfortunately, until quite recently not excited much general enthusiasm. It is now, however, becoming realised that the banishment of plague from Europe in the seventeenth century was in all probability bound up with altered conditions affecting prevalence of one or other species of vermin, and the practical extinction of typhus and relapsing fever in this country, dating from nearly fifty years ago . . . stands in close relationship with general improvement in the condition of the population as regards infection with body lice." Dr. Hamer's reasoned discussion on the flea hypothesis of the causation of scarlet fever gives much food for thought and will well repay careful study.

During the last nine years inquiries have been carried out in London with regard to the relationship between the flea curve and the curve of scarlet fever, and Dr. Hamer epitomises the result of his nine years' study in certain conclusions the most important of which are as follows :—(1) There is a very close correspondence in form between the last nine seasonal curves of scarlet fever and of flea prevalence. (2) Both scarlet fever and flea prevalence stand in close relationship with cycles of dry or wet years. (3) Study of school influence in scarlet fever leads to the conviction that some factor, other than immediate or direct personal infection from child to child, is concerned in maintaining the hold which the disease not infrequently obtains upon particular departments or class-rooms. (4) In striking contrast to dissemination of scarlet fever in homes and in schools stands the absence of any marked ability to spread in hospital wards. (5) Study of return cases shows that the influence of hospitalism has been unduly exaggerated. (6) Study of the geographical distribution of scarlet fever and of that of the human flea reveals a general correspondence in the present-day allocation of both. (7) The distribution, in time, of the fall in mortality from scarlet fever corresponds, so far as the facts can be ascertained, with diminution of prevalence of flea-bites in children. Dr. Hamer's interesting and instructive contribution to this subject will no doubt stimulate further investigation with regard to vermin as an ætiological factor in disease, and will bring about the wider realisation of the fact that uncleanliness and verminism constitute domestic insanitation and are a menace to public health. It is satisfactory, therefore, to note that the condition of bodily cleanliness of the intermediate age group of children and leavers examined since the year 1913 has improved by 7 per cent., and that it now stands at over 80 per cent.

### Defects and Minor Ailments.

The report contains full information regarding the prevalence of defects and minor ailments amongst the children examined, and the measures adopted for their correction and treatment. Dental caries exists, as in other districts, to a serious extent, but the figures given indicate that, while the children entering school show no improvement there is a steady increase in the number of children leaving school with sound teeth. Increased facilities have been provided during the year for dental treatment by the establishment of five new centres. The number of centres providing dental treatment at the end of the year was thirty-six, and of the 71,000 children requiring treatment 57,000 received treatment compared with 50,000 during 1916. Although there was a slight increase in the number of children referred for treatment for adenoids and enlarged tonsils as compared with 1916 the figures remain lower than in former years, and Dr. Hamer refers to the importance of systematic breathing exercises in securing improvement in these conditions. The examination of children for defective vision shows a higher incidence amongst girls than boys and that the number of boys and girls at the age of twelve having normal vision with or without glasses has decreased during the last two years; conditions inseparable from the war are regarded as in great measure responsible for this. Pulmonary tuberculosis was found in 0.1 per cent. of the children examined—a very low figure—but it has

* *Report of the Medical Officer of Health and the School Medical Officer of the Administrative County of London.*

☛ The previous article appeared on November 9, p. 113.

**London's Health**—(*continued*).

long been recognised that this disease may be present in young children in latent form without presenting any gross physical signs; in 0.2 per cent. tuberculosis in organs other than the lungs was found, but a larger percentage of both types of the disease was, however, found at urgent and special examinations. During the year 784 children suffering from tuberculosis were treated in residential institutions. It is satisfactory to note that there has been no increase in the incidence of nervous complaints notwithstanding existing conditions. Dr. Hamer states "there is no evidence that any portion of the strain of war conditions had been allowed to fall upon the children of school age generally in London."

A very excellent system of co-operation between the School Medical Service and the factory surgeon exists in London. The system provides that when a child on leaving school obtains work in a factory, the Labour Exchange should send to the City factory surgeon an abstract of the school doctor's report. By this means the benefit of school medical inspection is carried beyond the period of school life, and an organised effort is made to secure continuity of treatment and preventive measures so as to secure a higher standard of physical health and working capacity.

Dr. Hamer's report deals with many important problems relating to public and personal health. He emphasises by the continuity of his reasoned argument the intimate relationship between public health and the health of the school child, and the need for unified central control and administration. His report bears evidence to the high standard of work of the Public Health Service in London during the year, notwithstanding depleted staffs and the exigencies of the war.

